# Computational Model-Based Estimation of Mouse Eyeball Structure From Two-Dimensional Flatmount Microscopy Images

**DOI:** 10.1167/tvst.10.4.25

**Published:** 2021-04-23

**Authors:** Hongxiao Li, Hanyi Yu, Yong-Kyu Kim, Fusheng Wang, George Teodoro, Yi Jiang, John M. Nickerson, Jun Kong

**Affiliations:** 1 Department of Mathematics and Statistics, Georgia State University, Atlanta, GA, USA; 2 Institute of Biomedical Engineering, Chinese Academy of Medical Sciences & Peking Union Medical College, Tianjin, China; 3 Department of Computer Science, Emory University, Atlanta, GA, USA; 4 Department of Ophthalmology, Hallym University College of Medicine, Kangdong Sacred Heart Hospital, Seoul, South Korea; 5 Department of Computer Science, Stony Brook University, Stony Brook, NY, USA; 6 Department of Computer Science, Federal University of Minas Gerais, Belo Horizonte, Brazil; 7 Ophthalmology Department, Emory University, Atlanta, GA, USA

**Keywords:** retinal pigment epithelial, cell morphology, flatmount microscopy image, three-dimension sphere, eyeball modeling

## Abstract

**Purpose:**

Retinal pigment epithelial (RPE) cells serve as a supporter for the metabolism and visual function of photoreceptors and a barrier for photoreceptor protection. Morphology dynamics, spatial organization, distribution density, and growth patterns of RPE cells are important for further research on these RPE main functions. To enable such investigations within the authentic eyeball structure, a new method for estimating the three-dimensional (3D) eyeball sphere from two-dimensional tissue flatmount microscopy images was investigated.

**Methods:**

An error-correction term was formulated to compensate for the reconstruction error as a result of tissue distortions. The effect of the tissue-distortion error was evaluated by excluding partial data points from the low- and high-latitude zones. The error-correction parameter was learned automatically using a set of samples with the ground truth eyeball diameters measured with noncontact light-emitting diode micrometry at submicron accuracy and precision.

**Results:**

The analysis showed that the error-correction term in the reconstruction model is a valid method for modeling tissue distortions in the tissue flatmount preparation steps. With the error-correction model, the average relative error of the estimated eyeball diameter was reduced from 14% to 5%, and the absolute error was reduced from 0.22 to 0.03 mm.

**Conclusions:**

A new method for enabling RPE morphometry analysis with respect to locations on an eyeball sphere was created, an important step in increasing RPE research and eye disease diagnosis.

**Translational Relevance:**

This method enables one to derive RPE cell information from the 3D eyeball surface and helps characterize eyeball volume growth patterns under diseased conditions.

## Introduction

The retinal pigment epithelium (RPE) is the pigmented cell layer outside the neurosensory retina. This layer is firmly attached to the underlying choroid and the overlying retinal visual cells.^1^ In addition to providing nutrients to retinal visual cells, this cell layer performs numerous functions essential to the choroid and the photoreceptors,^2^ including scattered light absorption, transepithelial transport, spatial buffering of ions, maintenance of the visual cycle, phagocytosis of photoreceptor outer segments, and secretion of growth factors and immunosuppressive factors. Without RPE cells, photoreceptor cells die, and the choroid degenerates. Therefore RPE cells play an important role in maintaining retinal homeostasis and photoreceptor survival.

RPE aging is specifically involved in the pathogenesis of age-related macular degeneration (AMD).^3^^–^^8^ AMD is the leading cause of untreatable and irreversible central vision loss and legal blindness in industrialized countries.^9^^–^^12^ RPE cell morphological characteristics, such as shape, size, number, and geometric packing, provide informative features to determine RPE aging. For example, the size of an RPE cell in the fovea region is commonly smaller than that in peripheral areas,^4^^,^^13^ and the average RPE cell size increases with age.^13^^,^^14^ The RPE cell shape is closer to a regular hexagon in the central regions than in the outer zones.^15^^,^^16^ It is also known that the number of RPE cells decreases with age.^14^^,^^17^^–^^19^ RPE morphometries, such as cell density, eccentricity, form factor, and percent of hexagonal cells, also vary with aging and show significant topography-associated features.^19^^–^^21^ Therefore accurate quantification of the RPE cell morphometrics is essential for research and clinical diagnosis of AMD.

RPE flatmount^22^ microscopy has been used to quantitatively analyze RPE cell morphology.^4^^,^^6^^,^^18^^,^^19^^,^^21^^,^^23^ However, the experimental procedure for generating such a flatmount image usually introduces significant RPE cell distortion and loss, especially on the segmented lobe borders.^22^^,^^23^ This makes it difficult to measure RPE cell morphology on whole flatmount images accurately. In practice, regions not close to the lobe borders are selected for analysis.^21^^,^^23^ However, the morphometry features of whole tissue flatmount images would provide more informative insights into RPE cell histology. To achieve this, numerous digital image processing methods^24^^–^^31^ can be used to restore distorted or lost tissue regions in a RPE flatmount image. However, because of the considerable variations in RPE cell morphometry,^4^^,^^13^^,^^15^^,^^16^^,^^19^^,^^21^ accurate determination of the RPE cell locations on the three-dimensional (3D) eyeball model is a prerequisite for digital image inpainting and restoration analysis. The lack of methods for accurately locating tissue regions on a 3D eyeball structure makes the quantitative RPE cell morphometry analysis short of the anatomic and geographic interpretation. The geographic delineation of the 3D eyeball in current research pivots around the eyeball physiological structures. The common terminologies used to depict the geographic regions on the 3D eyeball surface include fovea, optic nerve head, perifovea, parafovea, and the peripheral areas from the central zones defined by the fovea or optic nerve head. The definitions of these locations of interest are imprecise and insufficient to support further investigations of RPE cell quantitative morphometrics. Furthermore, the quantification of tissue location–specific dynamics of RPE cell morphometry features, alignment orientation, and spatial organization are important for research and clinical investigations of AMD. Ideally, such investigations ought to be conducted in reference to authentic 3D eyeball structures. The loss of 3D eyeball structure information in two-dimensional (2D) tissue flatmount microscopy images presents a challenge for associating RPE cell features with the 3D eyeball anatomic architecture.

To address this challenge, we propose the use of a 3D sphere to model a 3D eyeball and a novel method for recovering the 3D eyeball sphere size and the corneal angle with 2D tissue flatmount images through the least-squares approach. The proposed method can effectively compensate for the estimation error as a result of tissue distortion and achieve a relative estimation error of 5.27% for the 3D eyeball size by automatically learning an error-correction term from the training dataset. The estimated 3D eyeball size can be used to reconstruct the 3D digital eyeball model and locate the tissue region on the 3D eyeball surface, providing accurate geographic information about the RPE areas based on latitude and longitude. This new technique enables promotion of further development of quantitative RPE cell morphometry analysis with respect to accurate eyeball anatomic locations. With tissue location in reference to the 3D eyeball surface determined by the proposed method, digital image inpainting and restoration analysis can be invoked to facilitate morphological analysis across the whole tissue flatmount image. Therefore the proposed new method for reconstructing the 3D eyeball sphere with 2D flatmount microscopy images can enhance our understanding of the relationships between RPE cell morphology and spatial organization and AMD histopathology, improving diagnosis and treatment of AMD and facilitating personalized medicine.

## Methods and Data

### Dataset

We estimated the sizes of 23 mouse eyeballs from different independent projects with mouse RPE flatmount microscopy images. The mouse eyeballs included 13 eyes from C57BL/6J mice ranging in age from postnatal day (P) 63 to P71 that were treated with laser photocoagulation to induce choroidal neovascularization, 2 eyes from C57BL/6J mice at P42, 3 eyes from 129/SV-E mice at P56 that were treated with sodium iodate tail vein injection, and 5 eyes of C57BL/6J mice at P104 from a control group without any specific treatment. The mice were handled according to the Association for Research in Vision and Ophthalmology (ARVO) guidelines, and the study was approved by the Emory University Institutional Animal Care and Use Committee. Mice were euthanized using CO_2_ asphyxiation before dissection. After the enucleation process, the eyeball was fixed in zinc formalin fixative (Z-fix; Anatech Ltd., Battle Creek, MI) for 10 minutes followed by washing with phosphate-buffered saline three times. The fixed eyeball was trimmed to remove extraocular fat and muscles before size measurement was performed. The mouse eyeball size was measured with a noncontact light-emitting diode (LED) micrometer (model 7030M with an LS-7601 controller and LS-H1W software; Keyence America Corp, Itasca, IL) as previously described.^32^ For five eyes from the control C57BL/6J mice, the eyeball size measurement was performed with a Keyence IM-6145 digital micrometer. The eyeball was placed in two different positions, that is, axial (anterior-posterior) and horizontal (equatorial), and the device automatically measured the diameter of each dimension. As we hypothesized that the eyeball is a sphere with a slight corneal bulge, the ground truth of the eyeball size was set with the equatorial diameter, that is, the average of the superior-inferior length and the nasal-temporal length. After the eyeball size measurement, the RPE flatmount was prepared with radial cuts from the center of the cornea back toward the optic nerve. The iris and the retina were removed with forceps, and additional incisions were made at the ciliary body and cornea margin to relieve tension from the sclera. Next, the RPE flatmounts were stained for rabbit anti-ZO1 antibody (1:100 dilution, catalog no. 61-7300; Invitrogen, Carlsbad, CA) and goat anti-rabbit immunoglobulin G (IgG) secondary antibody (catalog no. O11038; Invitrogen). The resulting flatmounts were imaged with a confocal imaging system (model C1; Nikon Inc., Melville, NY) with argon laser excitation at 488 nm. The confocal images were digitally merged (Adobe Photoshop CS2; Adobe Corp, San Jose, CA).

### Eyeball Spherical Reconstruction Model

**Figure 1.**
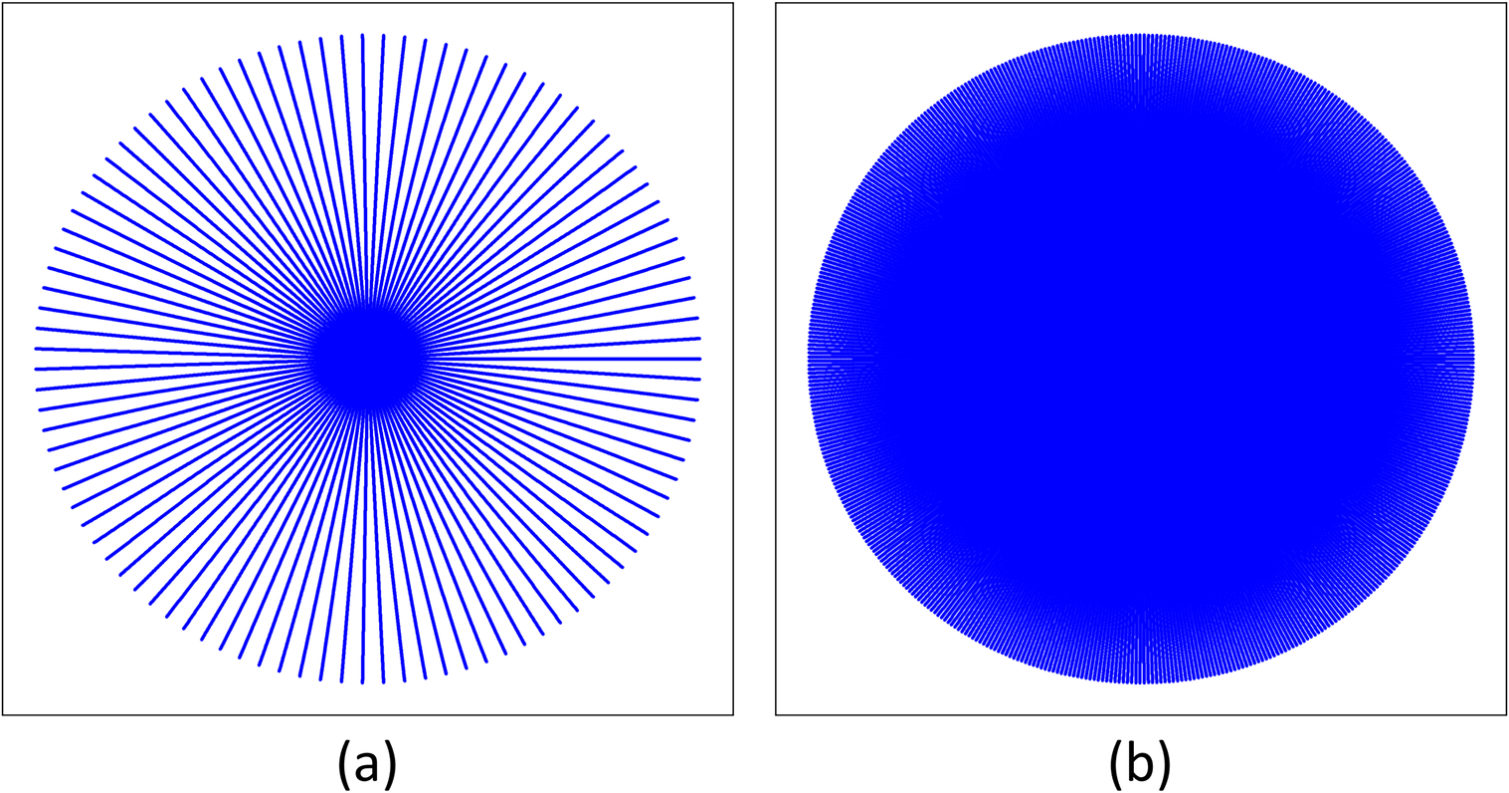
Schema of 2D flatmount images from 3D eyeballs after cutting and unfolding. Ideal flatmount images of a sphere cut into (a) 100 and (b) 500 meridians unfolded from the north pole with the tip on the south pole fixed, respectively, are shown.

A 3D mouse eyeball modeled with a 3D sphere is proposed. With this idealized model, we can have a set of meridians when the spherical shell is cut along lines of longitude. The more evenly cut the spherical shell, the more meridians this process produces. After the meridians are unfolded and pushed down from the sphere's north pole to the tangent plane at the south pole, the meridians become lines radiating from the sphere's south pole. On the resulting 2D flatmount image plane, the tips of such lines lie on a circle centered at the south pole. An “unfolding” result is illustrated in Figure 1. Under ideal circumstances, this circle is composed of the end points of infinite number of unfolded meridians extending radially from the south pole with a length of π × *R*, where *R* is the radius of the sphere.

Because of the physical limitations in flatmount practice, an eyeball can be cut only a limited number of times (usually 4, but no more than 8). The resulting meridians are radially distributed on the plane with the south pole as the common intersection point. We define this plane as the “south pole plane.” In addition, the latitude lines of the 3D sphere correspond to 2D concentric circles centered on the south pole in the resulting flat-mount plane. In the literature, it is common to refer to the cut “sectors” of the eyecup as “petals,” “lobes,” or “gores.” The reverse circumstance is a common engineering or cartography problem: a series of flat 2D patterns are cut to match 3D surfaces without wrinkles or gaps. For example, a 3D globe of Earth is assembled with a map on a 2D surface with 12 gores or sectors, and each sector corresponds to 30° of longitude. The gores are cut out and pasted on a sphere; and there are few wrinkles.^33^ Many other examples of the assembly of 3D bulging objects from 2D flat panels exist: parachutes, hot air balloons, tents, domes, corners in heating, ventilation, and air conditioning (HVAC) ductwork, and sails.

In this study, the reverse was performed. The eyecup was treated as a 3D spherical shell, cut into lobes or gores, and then flattened as much as possible, with the south pole of each gore touching at one point at the optic nerve head in the eye. The aim was to reduce the number of cuts to minimize damage to the RPE cells on the inner surface of the eyecup.

**Figure 2.**
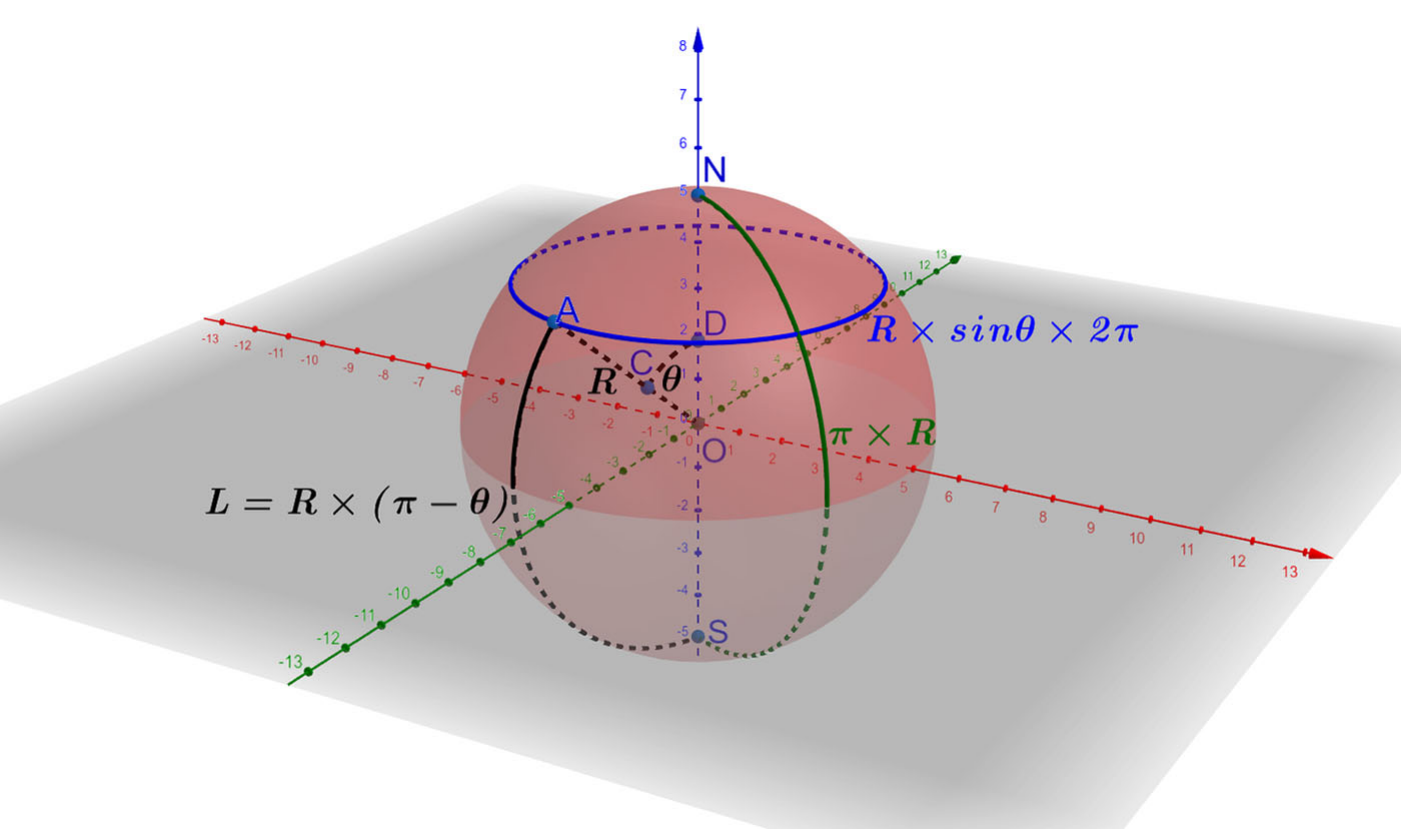
A schema of a 3D eyeball spherical model where *O*, *N*, and *S* are the center, north, and south poles of the sphere, respectively, are shown. Point *A* is an arbitrary point on the sphere surface. The resulting lines *ON* and *OA* form the angle θ. Point *C* and *D* are on the line *OA* and *ON* and equally distant from the origin *O*. South pole *S* of this spherical model corresponds to the optic nerve head; the top dome of the sphere represents the cornea. The length of line *OA* is the sphere radius *R*, whereas the length of the arc from *A* to *S* on the sphere is denoted as *L* = *R* × (π − θ) in black. Additionally, the circle perimeter of the top dome is *R* × sin θ ×  2π in blue; the length of the arc from the north pole *N* to the south pole *S* is π × *R* in green.

**Figure 3.**
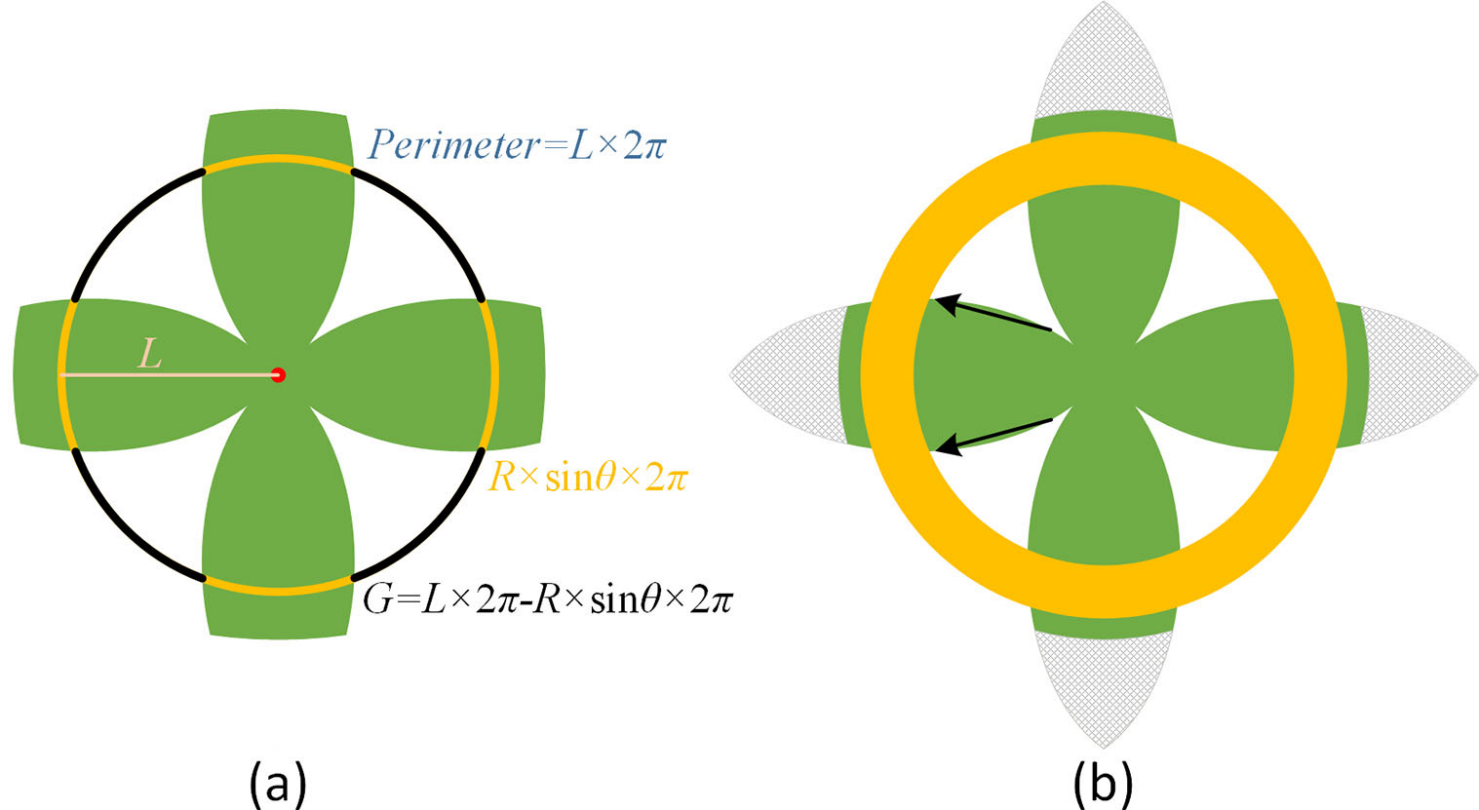
Schema of flatmount tissues on the south pole plane. (a) A schema in which the sphere is evenly cut into four lobes and pushed down to the south pole plane is shown. As an example of the concentric circles, the overlaid circle has radius *L* and perimeter *L* ×  2π. The yellow arcs represent the dome circle in Figure 2, with a length sum *R* × sin θ ×  2π. Black arcs indicate gaps between the neighboring lobes, and the resulting arc length sum is *G* = *L* ×  2π − *R* × sin θ ×  2π. (b) Solid arrows indicate the directions of the tissue distortions in the flattened lobe. The yellow ring indicates the equatorial zone of the sphere. The gray crosshatched areas represent the cornea components in the eyeball.

A schema of a 3D eyeball sphere model is shown in Figure 2, where *O* and *N* are the center and the north pole of the sphere, respectively. In Figure 2, angle θ is formed by lines *ON* and *OA* where *A* is an arbitrary point on the sphere. The south pole of this sphere model corresponds to the optic nerve head; the top dome of the sphere represents the cornea. In this coordinate system, the length of the shortest arc (i.e., the geodesic distance) from point *A* to south pole *S* on the sphere is *L* = *R* × (π − θ), where *R* is the sphere radius. To clarify terminology, a line of latitude for point *A* is “latitude *A*” and its corresponding concentric circle after projection on the south pole plane is “circle *A*”. The change in arc length during the flatmount process is assumed to be negligible. Therefore arc length *L* is identical to the radius of “circle *A*” on the 2D flatmount plane. With Figure 2, the length of “latitude *A*” can be computed as *R* × sin θ ×  2π. The perimeter of “circle *A*” on the south pole plane is *L* ×  2π. If the sphere is evenly cut into four lobes unfolded on the south pole plane, some gaps would occur between the neighboring lobes. This is illustrated in Figure 3(a). The sum of the lengths of all the gap arcs (*G*) on “circle *A*” is *G* = *L* ×  2π − *R* × sin θ ×  2π. From the previous discussion, *L* = *R* × (π − θ). Therefore the following equation governs the relationship across the gap arc length (*G*) in the 2D south pole plane, the geodesic distance from point *A* to the south pole on the sphere, and the sphere radius:

(1) $$G=2\pi \times \left(L-R\times \sin\left(\pi -\frac{L}{R}\right)\right)$$

The arguments *G* and *L* in Equation (1) can be measured from the flatmount images through image processing. Thus sphere radius *R* can be estimated with such measures through least-squares curve fitting.^34^

### Tissue Distortion Modeling

For production of flatmount images, the dissected tissue lobes are flattened on the south pole plane, which is the surface of a microscope slide. As the tissue lobes originally cover the surface of a sphere, the flattening procedure introduces tension and compression within the tissue lobes and leads to some tissue distortion. The zones a lobe covers in the northern latitudes tend to need an additional, relaxing dissection cut for them to lie flat, which introduces an additional gap. Given the location-invariant tissue elasticity, the lobe tissue arc lengths before and after dissections are approximately constant, whereas the interlobe gaps vary substantially according to the latitude, much like 2D map projections in geographic representations of Earth on a globe.^35^

A collective set of forces, in aggregate, a “flattening force,” push the lobes flat on the glass slide. The flattening force includes gravity, surface tension, tamping forces of the dissecting tools, and force caused by the downward pressure of the overlying coverslip. These forces are resisted by a cohesive force in the tissues. However, there is no resistive force at the cuts, allowing the splayed eyecup to lie approximately flat.

**Figure 4.**
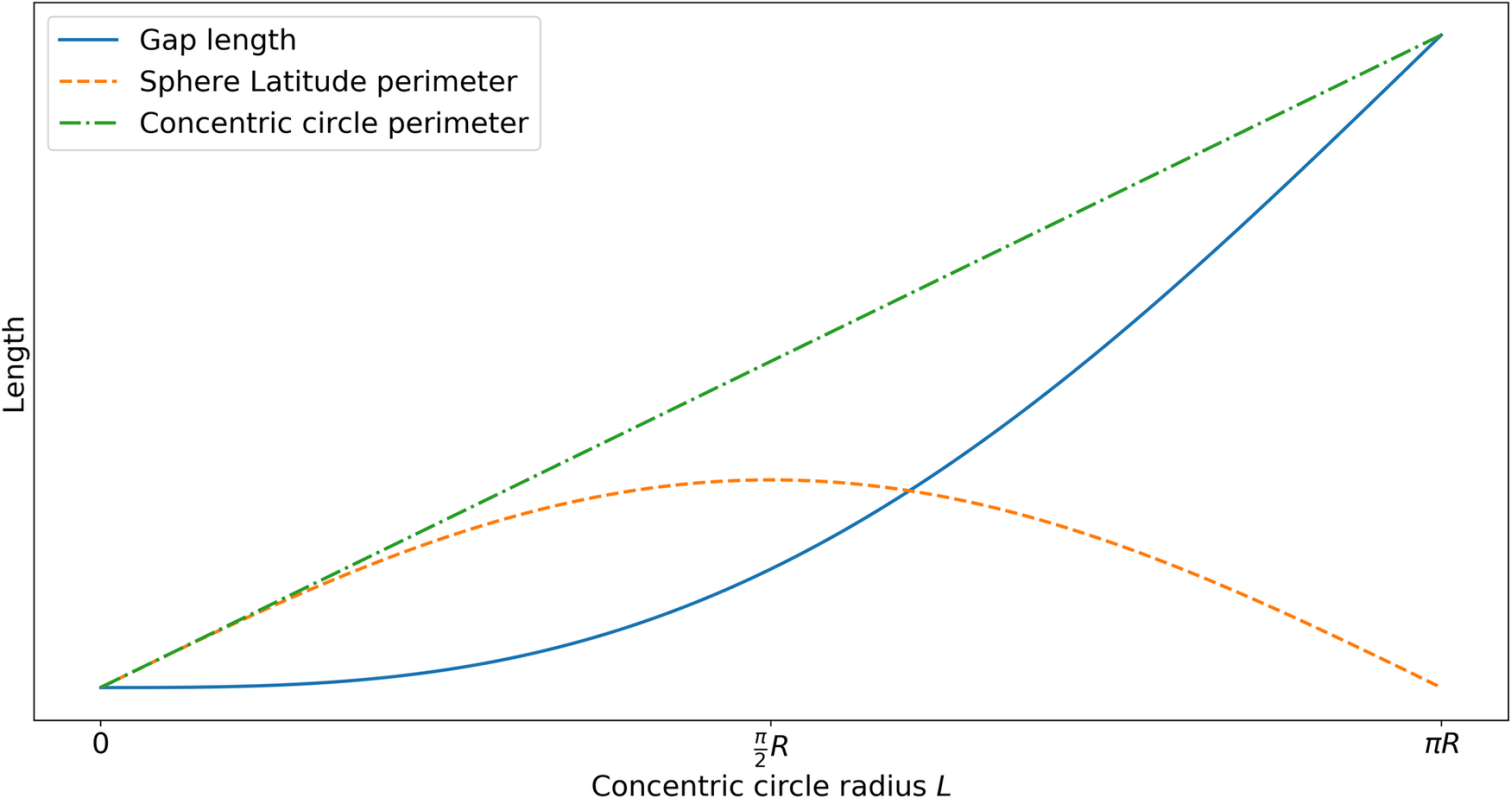
A plot illustrating the change between the sphere latitude perimeter and the concentric circle perimeter. Given *R* is the sphere radius, the sphere latitude perimeter (red), the concentric circle perimeter (green), and their difference (i.e., the gap length in blue) as a function of concentric circle radius *L* are plotted. The sphere latitude perimeter reflects tissue cohesion, whereas the concentric circle perimeter suggests the effect of the flattening force.

**Figure 5.**
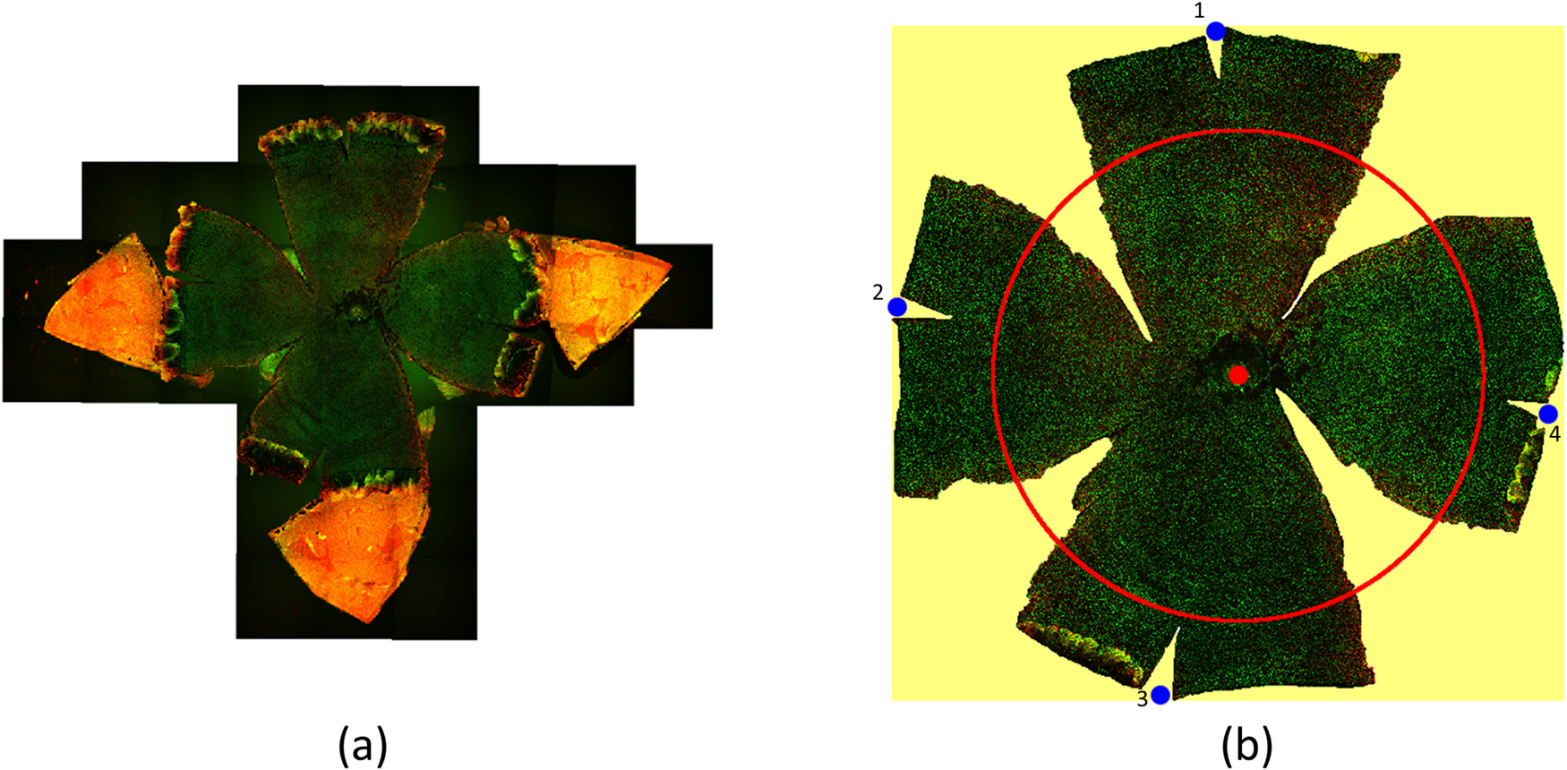
Flatmount microscopy image analysis and measurement. (a) A typical flatmount microscopy image is shown. The orange component at the tip of each lobe corresponds to the cornea in the northern hemisphere. (b) A processed image is shown. The flatmount image is transformed into the hue, saturation, value (HSV) color space, and Otsu's threshold is applied to the saturation channel for differentiation of the background regions in yellow and the foreground tissue regions. An example concentric circle is illustrated in red. The number of pixels on the red circle over the background region in yellow is counted to measure the length of the gap between lobes *G*. A red point on the optic nerve head is annotated at the origin of the concentric circles. The four blue points (i.e., lobe-marker points) are used to determine the middle axis lengths of four lobes: *M*_1_, *M*_2_, *M*_3_, and *M*_4_.

Although the flattening procedure inevitably changes the relative cell locations and the associated radiating meridians, tissue cohesion resists such deformation, resulting in gaps between the lobes. The gap between two lobes at any one point is small near the south pole and larger everywhere else, depending on the location of the point. The relationships among the flattening effect, tissue cohesion, and the size of the gap where dissection cuts were made are shown in Figure 4, where the sphere latitude perimeter is compared with the concentric circle perimeter. The sphere latitude perimeter reflects the uncut tissue, whereas the concentric circle perimeter indicates the outcome of the flattening force that splays out the lobes of the cut eyecup. The net effect of these two factors determines how the spherical shell of the RPE-choroid-sclera were deformed into several flat lobes. The two perimeters increase with the increase in *L* in the southern hemisphere; that is, *L* ≤ *R* × π/2. On the northern hemisphere (i.e., *L* > *R* × π/2), the tissue width starts to decrease with the increase in *L*. By contrast, the gap arc length continues to increase. In higher-latitude zones of the northern hemisphere, the gap arc length between lobes is much larger than the tissue lobe widths. Ultimately, at the tips of the lobes, tissue vanishes, and the gaps close to become a continuous circle with radius *R* × π. The cut lobes may have irregular shapes in any zone (or latitude) because of the difficulty of the dissection, as shown in Figure 5(a). However, the proposed model as depicted by Equation (1) is fully applicable regardless of latitude or hemisphere.

Tissues off the lobe middle axis are subject to some distortion after the flattening process with the distortion directions shown in Figure 3(b). Because of tissue deformations, the measured length of the gaps between the lobes in the flatmount image can be different from that of the ideal case described by Equation (1). Therefore an error term *E* is introduced to compensate for this tissue distortion effect:

(2) $$G-E=2\pi \times \left(L-R\times \sin\left(\pi -\frac{L}{R}\right)\right)$$

Given the location-invariant tissue elasticity, the extent of the tissue distortion on each concentric circle is approximately proportional to the tissue mass on that circle. Therefore the error term is designed to be positively correlated with sin θ, and defined as *E* = *k* × sin θ, where *k* is an unknown tissue distortion coefficient that depends on how 3D eyeball tissues are flattened to the 2D flatmount. With this error term, the proposed model considering the tissue deformation effect becomes:

(3) $$G=2\pi \times \left(L-\left(R-\frac{k}{2\pi }\right)\times \sin\left(\pi -\frac{L}{R}\right)\right)$$

### Flatmount Image Analysis and Measurement

The parameters *G*, *L*, and *k* in Equation (3) must be known before sphere radius *R* can be estimated. The length of the gaps between lobes *G* and the radius of the concentric circle on the 2D flatmount plane *L* can be directly derived from the flatmount image by counting the number of pixels with image processing methods. The tissue distortion coefficient *k* can be determined with Equation (3) using training samples with known ground truth *R*.

As the corneal section of an eyeball does not contain any visual neurons, it was cut before flatmount images were produced. Moreover, it was easier to flatten the cut lobes onto the south pole plane (i.e., the microscope slide) after the corneal section was removed during the tissue preparation procedure. The resulting flatmount images were transformed into the hue, saturation, value (HSV) color space, and Otsu's threshold was applied to the saturation channel for differentiation of the background and foreground tissue regions. The detected background region is illustrated in Figure 5(b) with yellow. The red point is the origin of the concentric circles. The four blue points are selected as the distal points on the lobe middle axis and called the lobe-marker points. They are used to determine the middle axis lengths of four lobes: *M*_1_, *M*_2_, *M*_3_, and *M*_4_. The middle axis length is the distance from the origin to a lobe-marker point. In this implementation, Aperio ImageScope V12.3.3^36^ was used to mark these critical marker points manually on the flatmount images.

Next, a set of concentric circles that are 1-pixel thick were drawn centered on the origin. The value of this circle radius was derived from an arithmetic array *L_Arr_* ∈ [1,  *M_min_*], where $M_{min}=\min_{n}\left\{M_{n}\right\},\;n=1,2,3,4$. For each circle with the radius *L_i_* ∈ *L_Arr_*, the number of circle pixels in the background region was counted to measure the length in-between lobe gaps *G_i_* ∈ *G_Arr_*. The length of the arithmetic array was set to be 500 in the implementations. The maximum of the lobe middle axis length $M_{max}=\max_{n}\left\{M_{n}\right\},\;n=1,2,3,4$ was used to estimate corneal angle θ after sphere radius *R* was estimated.

### Estimation of Tissue Distortion Coefficient, Sphere Radius, and Corneal Angle

As an unknown tissue distortion coefficient *k* was introduced in Equation (3) to correct the tissue distortion error, it was necessary to estimate *k* before we could calculate sphere radius *R* and corneal angle θ. The error-correction term can be learned from a number of samples with known ground truth *R* by the least-squares algorithm in the model. As all tissues were prepared similarly, the resulting tissue distortion coefficients were similar. As one tissue distortion coefficient *k* was estimated from each such sample with a known *R*, the mean of the tissue distortion coefficients was taken as the learned *k* for further *R* estimation. To solve *R* from Equation (3) with the least-squares method, the optimization process was prevented from converging to a trivial solution (i.e., *R* ≈ 0) by setting the initial value for *R* much larger than 0, (e.g., 100). With the estimated *R*, the corneal angle θ can be readily found as:

(4) $$\theta =\pi -\frac{L}{R}$$

where $L=\max_{n}\left\{M_{n}\right\},\;n=1,2,3,4$. Additionally, Equation (4) can be used to calculate the latitude of any 2D flatmount region on the 3D eyeball sphere surface. In this case, *L* is the radius of the corresponding concentric circle measured with the 2D flatmount image.

## Results

### Investigations of Estimation Error by Distinct Latitude Zones

**Figure 6.**
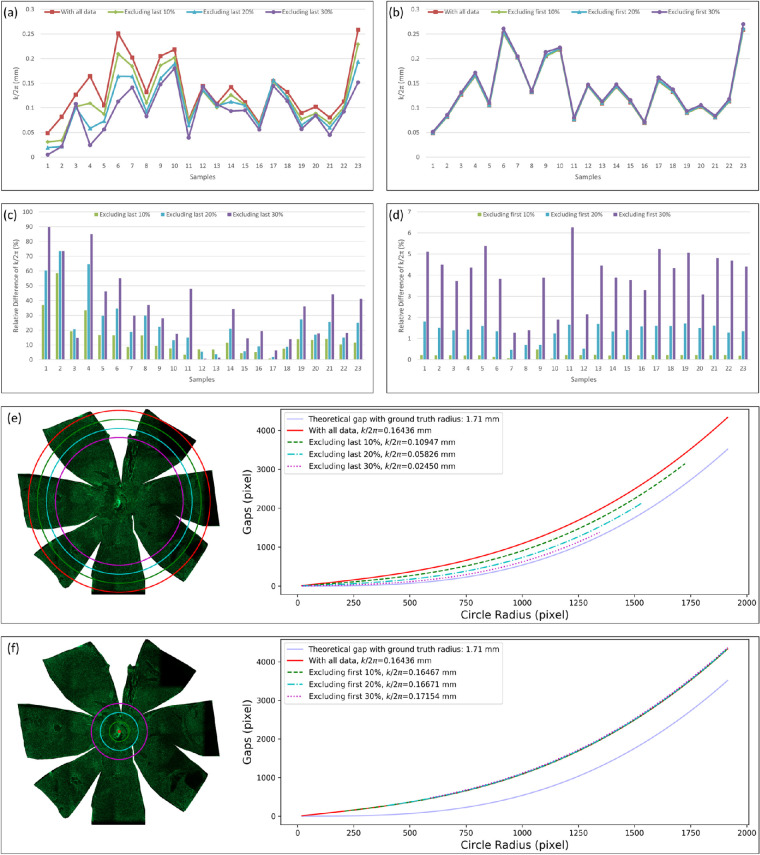
Estimates of the tissue distortion coefficient *k* based on full and partial data points. The estimates of tissue distortion coefficient *k* are plotted from 23 samples based on the full dataset and partial data points excluding 10%, 20%, and 30% of data points from (a) the low-latitude zones and (b) the high-latitude zones. Differences in tissue distortion coefficient estimate *k* between the full data point set and the partial dataset with partial data point removal from the (c) low-latitude and (d) high-latitude zones. A typical tissue flatmount image is shown with its fitting curves associated with tissue distortion coefficient *k* estimated with the full dataset and partial data points not including some from the (e) low-latitude and (f) high-latitude zones. These fitting curves are associated with tissue distortion coefficient *k* estimated with distinct data point sets: (blue solid) theoretic tissue interlobe gaps from Equation (1) with the ground truth radius; (red solid) full dataset; (green dashed) partial data points with 10% of data points excluded; (blue dash-dotted) partial data points with 20% of data points excluded; (purple dotted) partial data points with 30% of data points excluded.

To systematically investigate estimation error sources, the error effect of the data points from the low- and high-latitude zones was qualitatively analyzed. Evaluating the error effect from the low-latitude zones, the tissue distortion coefficient *k* was estimated based on Equation (3) with data points excluding those from the low-latitude zones. In the evaluations, the last 10%, 20%, and 30% of data points (i.e., 50, 100, and 150 data points from the 500 data points) in close proximity to the low-latitude zones were removed from *L_Arr_* and *G_Arr_*, respectively. Similarly to test the error effect from the high-latitude zones, the tissue distortion coefficient *k* was estimated based on Equation (3) with data points excluding partial data from the high-latitude zones. In the evaluations, the first 10%, 20%, and 30% of data points (i.e., 50, 100, and 150 data points from the 500 data points) in close proximity to the high-latitude zones were removed from *L_Arr_* and *G_Arr_*, respectively. The resulting estimates of the tissue distortion coefficient *k* from 23 samples based on the full dataset and partial data points excluding some from the low- and high-latitude zones are shown in Table 1 and Figure 6(a-b), respectively. Additionally, partial data points were removed from the low- and high-latitude zones, and the resulting deviations in the estimated tissue distortion coefficient *k* from the reference estimate with the full data point set are shown in Figure 6(c-d). The fitting curves associated with the tissue distortion coefficients estimated with distinct data point sets excluding some from the low- and high-latitude zone in one typical sample are shown in Figure 6(e–f).

**Table 1.** Estimates of the Tissue Distortion Coefficient *k* With Full Data Points and Partial Data Points Excluding Some From the Low- and High-Latitude Zones

Data	Averaged *k*/2π (mm)
All data points	0.1355
90% of data points (excluding 10% from the low-latitude zones)	0.1176
80% of data points (excluding 20% from the low-latitude zones)	0.1046
70% of data points (excluding 30% from the low-latitude zones)	0.0915
90% of data points (excluding 10% from the high-latitude zones)	0.1357
80% of data points (excluding 20% from the high-latitude zones)	0.1373
70% of data points (excluding 30% from the high-latitude zones)	0.1406

The estimate of tissue distortion coefficient *k* decreased as more data points from the low-latitude zones were removed as shown in Figure 6(a). Additionally, the estimated *k* shown in Figure 6(b) remained at about the same value as more data points were removed from the high-latitude zones. The difference between the tissue distortion coefficient *k* estimated with the full and distinct partial data point sets was further computed. The relative difference by percentage for each sample is plotted in Figure 6(c) and (d) in which partial data were removed from low- and high-latitude zones, respectively. It is noticeable that the tissue distortion coefficient dropped significantly when more data points were removed from the low-latitude zones for most samples shown in Figure 6(c), whereas the tissue distortion coefficient did not change significantly (<5%) when more data points were removed from the high-latitude zones for the vast majority of the samples shown in Figure 6(d). Furthermore, the same patterns were observed with the curve fitting results shown in Figure 6(e) and (f) . In Figure 6(e), the interlobe gap-fitting curves associated with distinct tissue distortion coefficients deviate by a larger decrease from the theoretic tissue interlobe gaps from Equation (1) as more data points were removed from the low-latitude zones. In contrast, all interlobe gap-fitting curves associated with distinct tissue distortion coefficients were close to each other when partial data points were removed from the high-latitude zones. The difference in the error compensation term *k*/2π estimated between all and partial data points for each sample was computed. The resulting variances of the error compensation term difference associated with different partial datasets are presented in Table 2. The variances with partial data excluded from the low-latitude zones were consistently larger than those estimated with partial data excluded from the high-latitude zones by at least three orders of magnitude.

**Table 2.** The Variance of Difference in the Error Compensation Term *k*/2π Estimated Between All and Partial Data Points With Some Data Points Removed From the Low- and High-Latitude Zones

Removed Data Point Percentage	Data Removed From Low-Latitude	Data Removed From High-Latitude
10%	1.94E-04	7.78E-08
20%	7.29E-04	5.49E-07
30%	1.46E-03	5.81E-06

All these results suggest that the data points from the low-latitude zones contributed more to the gap error than those from the high-latitude zones in the southern hemisphere. These experimental results also corroborated our hypothesis that the local interlobe tissue gap error *E* due to tissue distortion in Equation (2) increases when the latitude decreases, suggesting the equatorial zones have larger gap errors than the polar zones. Based on this conclusion that the low-latitude zones have a larger impact on gap error than the high-latitude zones, data points from the low-latitude zones have a more influential impact on the gap-radius fitting results than the same number of data points from the high-latitude zones. Additionally, we can conclude that the interlobe gap error is more sensitive to the manual artifacts in low-latitude zones than in high-latitude zones during the tissue flatmount preparation procedure.

### Estimation of 3D Eyeball Sphere Radius *R* and Corneal Angle θ

The 3-fold, 5-fold, and leave-one-out (LOO) cross-validation (CV) strategies were used to evaluate the performance of the model described in Equation (3) for the estimation of the eyeball radius. The CV process divided the dataset into training and testing samples with different strategies. The training samples were used to estimate the tissue distortion coefficient *k* in Equation (3). The mean of the estimated tissue distortion coefficients was computed for estimating the eyeball radius of the testing samples. Additionally, the estimates from the non-error-correction model described in Equation (1) were used as results from a control group.

**Figure 7.**
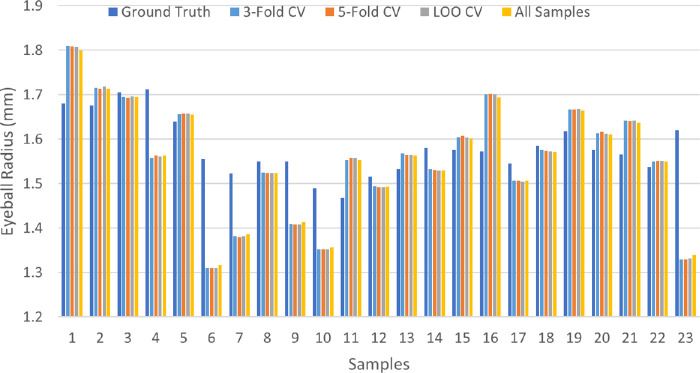
Estimates of eyeball radius with distinct tissue distortion coefficient strategies. The eyeball radius estimates from distinct learning methods are shown for the tissue distortion coefficient and compared with the eyeball ground truth: three-fold CV, five-fold CV, LOO-CV, and the all-sample strategy (i.e., the tissue distortion coefficient learned from 23 testing samples applied to the same 23 testing samples).

The 3-fold CV randomly partitioned the 23 samples into 3 portions with 8, 8, and 7 samples, respectively. The 5-fold CV randomly partitioned the dataset into 5 portions with 5, 5, 5, 4, and 4 samples, respectively. With 3-fold and 5-fold CV experiments, the tissue distortion coefficient was learned from all but 1 portion, and the learned tissue distortion coefficient was used to estimate the eyeball sphere radius with Equation (3). The 3-fold and 5-fold CVs were implemented 20 times with different random generators, respectively. The LOO-CV split the 23 samples 23 times. Each time, one sample was left out as the testing sample, and the remaining 22 samples were used to learn the tissue distortion coefficient. After the CVs were repeated 20 times, there were 20 estimated eyeball sphere radius values for each sample with the 3-fold and 5-fold CV experiments, respectively. Some descriptors, such as the minimum, maximum, median, and mean, of the sample-wise radius estimate standard deviation with 3-fold and 5-fold CV experiments are presented in Table 3. Results in Table 3 show that the sample-wise radius estimate standard deviation from the 3-fold and 5-fold CV are much smaller than the individual eyeball radius that ranged from 1 to 2, suggesting 20 estimates for each sample were closely scattered around the mean. Therefore it validated the use of the average estimate to represent the 20 estimates of each sample in the 3-fold and 5-fold CV experiments. The estimated eyeball radius is shown with the CV methods in Figure 7. The difference in the radius estimates from the different CV methods was negligible. Therefore the LOO-CV estimates were used to represent the eyeball radius. In addition to the three CV methods, the tissue distortion coefficient with all samples, that is, all-sample strategy, was learned. The fact that estimates from all three CV strategies were similar to those from the all-sample strategy demonstrates the strong robustness of the proposed model described in Equation (3).

**Table 3.** Standard Deviation of the Estimated Eyeball Radius of the 23 Samples With the 3-Fold and 5-Fold CV

Sample Index	3-Fold CV	5-Fold CV
1	1.43E-02	1.02E-02
2	1.77E-02	1.34E-02
3	1.24E-02	9.71E-03
4	1.61E-02	8.50E-03
5	1.60E-02	9.80E-03
6	1.11E-02	6.98E-03
7	1.27E-02	8.30E-03
8	1.34E-02	9.25E-03
9	1.00E-02	7.22E-03
10	9.25E-03	6.44E-03
11	1.72E-02	1.26E-02
12	1.29E-02	9.12E-03
13	8.47E-03	9.31E-03
14	1.56E-02	9.64E-03
15	8.89E-03	9.15E-03
16	1.60E-02	1.01E-02
17	1.20E-02	8.47E-03
18	1.56E-02	9.17E-03
19	1.33E-02	6.19E-03
20	1.10E-02	8.77E-03
21	1.63E-02	1.05E-02
22	1.49E-02	8.41E-03
23	1.16E-02	7.11E-03
Minimum	8.47E-03	6.19E-03
Maximum	1.77E-02	1.34E-02
Median	1.33E-02	9.15E-03
Mean	1.33E-02	9.06E-03

**Figure 8.**
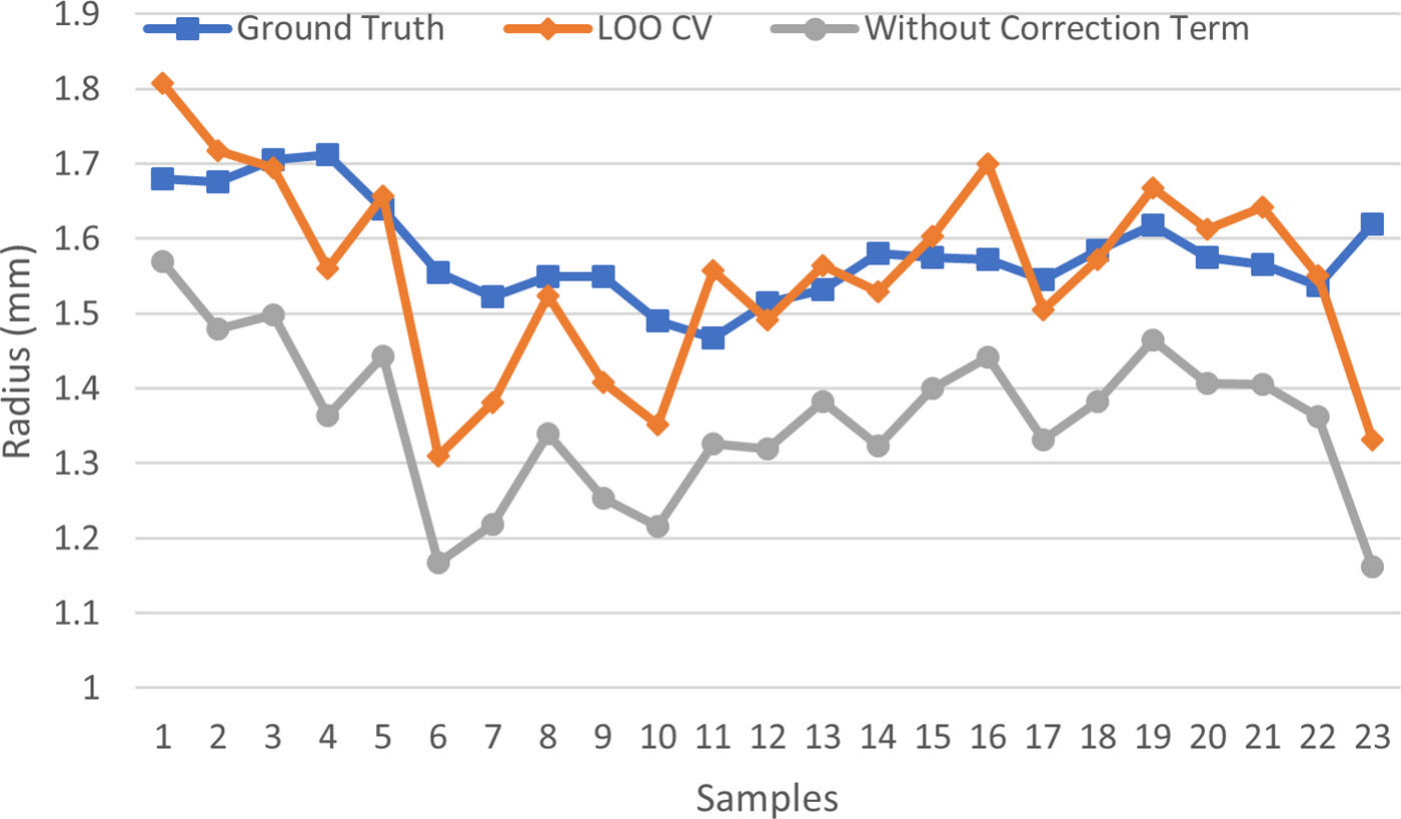
Radius comparison with the ground truth, the radius estimated with the tissue distortion coefficient using the LOO-CV strategy, and the estimate from the model without the tissue deformation correction term.

**Figure 9.**
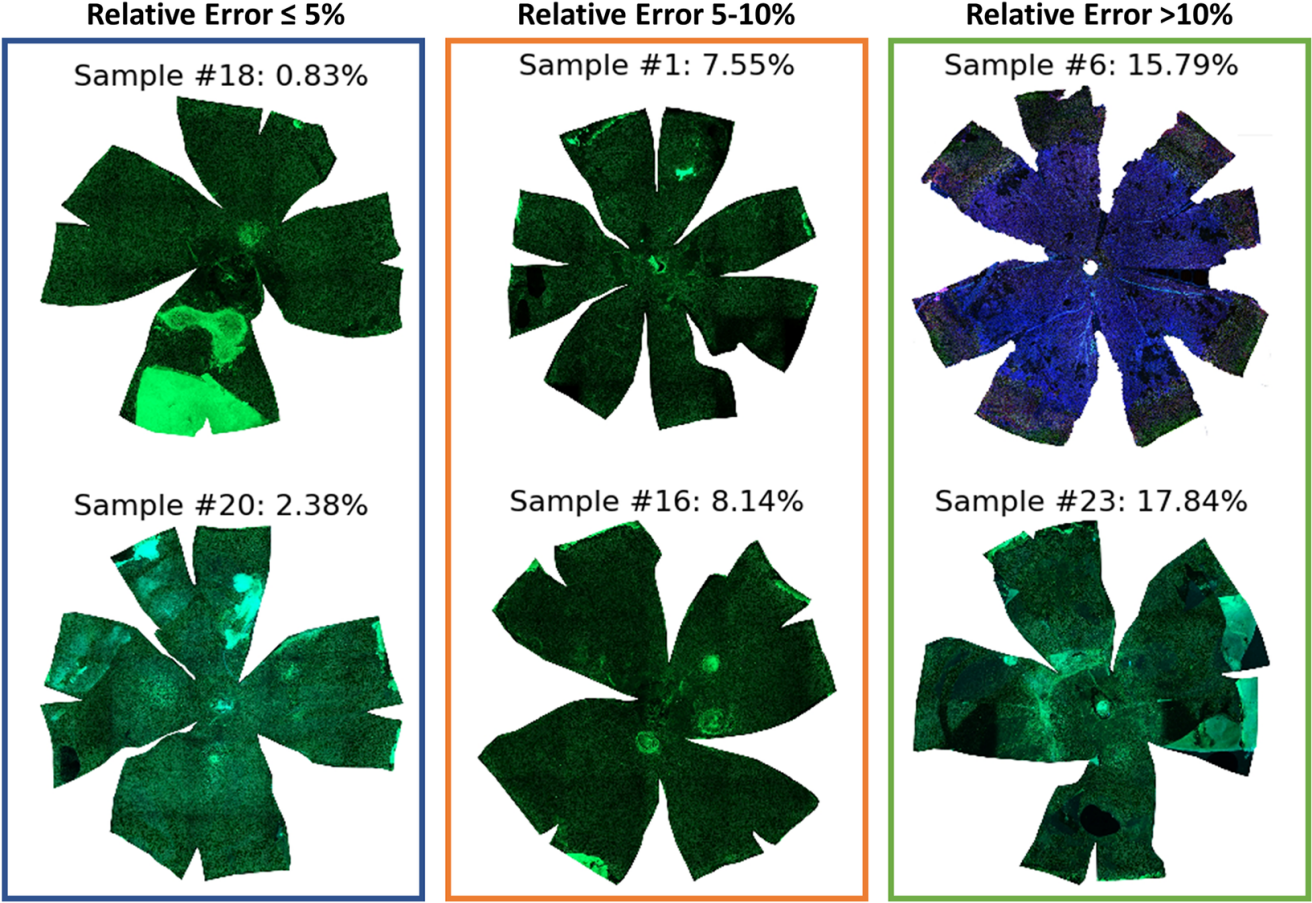
Representative tissue flatmount microscopy image examples from three classes are illustrated with the relative error: (left column) ≤5%, (middle) 5%–10%, and (right) >10%. Two flatmount microscopy image samples from each class are shown. The sample number and the relative error are above each sample image.

The eyeball radius ground truth was plotted and compared with the estimates with the tissue distortion coefficient from the LOO-CV strategy, shown in Figure 8. With the relative radius difference percentage, the 23 samples were divided into 3 estimation accuracy classes in Table 4: (1) less than or equal to 5%, (2) larger than 5% but less than or equal to 10%, and (3) larger than 10%. Of these 23 samples, 14 samples (i.e., 60.87%) were in the first class, with less than 5% error. The second class (greater than 5% but less than 10% error) had 7 samples (30.43%). Only 2 samples had more than 10% error (i.e., 15.79% and 17.84%, respectively). The average radius difference and the relative radius difference percentage were 0.03 mm and 5.27%, respectively. Two tissue flatmount microscopy image examples from each class are shown in Figure 9.

**Table 4.** The Difference and the Relative Difference Percentage With the Eyeball Radius Ground Truth and Estimates by Our Model Described in Equation (3)

Sample Index	Radius Difference (mm)	Relative Radius Difference Percentage (%)	Estimation Accuracy Class
3	0.01	0.57	Class #1: [0%, 5%] 14 samples
18	0.01	0.83	
22	–0.01	0.89	
5	–0.02	1.12	
12	0.02	1.55	
8	0.03	1.67	
15	–0.03	1.83	
13	–0.03	2.06	
20	–0.04	2.38	
2	–0.04	2.52	
17	0.04	2.61	
19	–0.05	3.08	
14	0.05	3.23	
21	–0.08	4.90	
11	–0.09	6.17	Class #2: (5%, 10%] 7 samples
1	–0.13	7.55	
16	–0.13	8.14	
4	0.15	8.85	
9	0.14	9.14	
7	0.14	9.24	
10	0.14	9.28	
6	0.25	15.79	Class #3: (10%, 20%] 2 samples
23	0.29	17.84	
**Mean**	**0.03**	**5.27**	

LOO-CV method is used to learn the tissue distortion coefficient. By the estimate relative difference percentage, the 23 samples are divided into three estimation accuracy classes based on the relative difference percentage.

In Figure 8, the radius estimates from the error-correction model described by Equation (3) and the ideal model described in Equation (1) are compared. The radius difference and the relative radius difference percentage between the ground truth and the estimates of the 23 samples are shown in Table 5. The resulting average radius difference and relative radius difference percentage were 0.22 mm and 14.05%, respectively. The average radius difference from the error-correction free model was one order of magnitude higher than that from the proposed model considering tissue deformation described in Equation (3). Additionally, the relative difference percentage from the error-correction free model was nearly three times as large as that from the error-correction model. These results suggest the salient effectiveness of the error-correction model described in Equation (3).

**Table 5.** The Difference Between the Eyeball Radius Ground Truth and Estimates by Our Model Described in Equation (1) Without Tissue Deformation Correction Term, and the Resulting Relative Difference Percentage

Sample No.	Radius Difference (mm)	Relative Radius Difference Percentage (%)
1	0.11	6.58
16	0.13	8.34
19	0.15	9.46
11	0.14	9.63
13	0.15	9.81
21	0.16	10.17
20	0.17	10.64
15	0.17	11.10
22	0.17	11.36
2	0.20	11.69
5	0.20	11.92
3	0.21	12.09
18	0.20	12.76
12	0.19	12.87
8	0.21	13.55
17	0.21	13.83
14	0.26	16.21
10	0.27	18.40
9	0.30	19.13
7	0.30	19.99
4	0.35	20.37
6	0.39	24.97
23	0.46	28.27
**Mean**	**0.22**	**14.05**

Finally, corneal angle θ was calculated with Equation (4) after eyeball sphere radius *R* was estimated. The estimated corneal angle θ can help characterize tissue surface coverage on the 3D eyeball sphere, and this measurement is helpful in analyzing microphthalmia, profound myopia or hyperopia, and many other eye diseases. The estimated radius *R* and corneal angle θ from 23 samples are shown in Table 6. Additionally, the estimates using the LOO-CV strategy for learning the tissue deformation coefficient are shown in Supplementary Figure S1.

**Table 6.** Eyeball Sphere Radius and Corneal Angle θ Estimated From the Error-Correction Model by Equation (3) Using 3-Fold, 5-Fold, LOO-CV, and the Error-Correction Free Model by Equation (1) are Presented

Sample Index	Ground Truth Radius	3-Fold CV Radius	5-Fold CV Radius	LOO CV Radius	Correction Free Model Radius	3-Fold CV Theta	5-Fold CV Theta	LOO CV Theta	Correction Free Model Theta
1	1.68	1.81	1.81	1.81	1.57	91.35	91.32	91.27	77.85
2	1.68	1.72	1.71	1.72	1.48	85.77	85.66	85.90	70.75
3	1.71	1.70	1.69	1.70	1.50	84.97	84.83	84.99	72.54
4	1.71	1.56	1.56	1.56	1.36	81.46	81.80	81.66	67.43
5	1.64	1.66	1.66	1.66	1.44	86.21	86.30	86.32	72.47
6	1.56	1.31	1.31	1.31	1.17	72.64	72.65	72.61	59.48
7	1.52	1.38	1.38	1.38	1.22	76.50	76.29	76.51	62.62
8	1.55	1.52	1.52	1.52	1.34	80.21	80.18	80.21	66.50
9	1.55	1.41	1.41	1.41	1.25	65.54	65.46	65.48	51.33
10	1.49	1.35	1.35	1.35	1.22	64.04	64.06	64.06	51.10
11	1.47	1.55	1.56	1.56	1.33	87.57	87.84	87.89	71.79
12	1.52	1.49	1.49	1.49	1.32	79.32	79.23	79.22	66.12
13	1.53	1.57	1.56	1.56	1.38	82.77	82.58	82.55	69.72
14	1.58	1.53	1.53	1.53	1.32	86.61	86.47	86.38	71.89
15	1.58	1.60	1.61	1.60	1.40	83.62	83.81	83.61	69.59
16	1.57	1.70	1.70	1.70	1.44	83.91	83.94	83.89	66.62
17	1.55	1.51	1.51	1.50	1.33	78.22	78.27	78.15	64.89
18	1.59	1.58	1.57	1.57	1.38	81.74	81.62	81.54	68.08
19	1.62	1.67	1.67	1.67	1.46	88.08	88.09	88.12	75.39
20	1.58	1.61	1.62	1.61	1.41	85.04	85.22	85.01	71.17
21	1.57	1.64	1.64	1.64	1.41	89.18	89.13	89.21	73.98
22	1.54	1.55	1.55	1.55	1.36	83.50	83.63	83.66	70.35
23	1.62	1.33	1.33	1.33	1.16	65.01	65.04	65.17	48.47

The units for radius and corneal angle are millimeter and degree, respectively.

### Software Interface Development

This modeling and estimation method was implemented in Python. Users provide annotations on the critical marker points, such as the concentric circle origin, lobe-marker points in Figure 5(b), for each flatmount image using Aperio ImageScope,^36^ save the annotation file in the image folder and invoke the software to process all images in a batch mode. To further promote software dissemination and usage, a graphic user interface (GUI) is provided in MATLAB (MathWorks Inc., Natick, MA). The MATLAB GUI integrates the manual labeling process with the computational estimation process and makes it easier to use the software. The user interfaces for estimation of the tissue distortion coefficient and the eyeball size are shown in Supplementary Figure S2. The interface on the top was developed to estimate the tissue distortion coefficient *k* using sample flatmount images with known size. With the “Load” button, users can load an Excel file (Microsoft Corp., Redmond, WA) with the following information: image name, image path, ground truth eyeball size, and unit conversion ratio (i.e., microns per pixel). Within this interface, users can then mark the concentric circle origin with the “Set” button and lobe-marker points with the “New” button for each image. After all images are marked, the interface can be used to estimate the tissue distortion coefficient for each image and export results to an Excel file with the “Export” button. The graphic interface at the bottom is used to estimate the eyeball diameter for each flatmount image with the learned tissue distortion coefficients. It loads the learned tissue distortion coefficients in the previous step and computes the average tissue distortion coefficient. Additionally, it loads an Excel file that includes information about the image names, image path, and unit conversion ratio (i.e., microns per pixel). Users use this interface to mark the concentric circle origin and lobe-marker points and export another Excel file containing estimated diameters for eyeballs.

## Discussion

RPE cell morphology, mass orientation, and spatial organization play a vital role in better characterizing and understanding RPE histopathology and physiology.^21^^,^^37^^–^^39^ Despite extensive investigations on this topic, current quantitative RPE cell analyses with histology slides lack accurate eyeball geographic location information. Analysis of RPE cells without reference to their geographic locations is a bottleneck in understanding RPE cell organization. To address this challenge, a novel modeling method for recovering individual 3D eyeball size from a 2D tissue flatmount microscopy image was proposed in this study. Such a technical method enables RPE features extracted from a 2D flatmount image to be mapped to a recovered 3D eyeball structure, allowing better insights according to their 3D locations.

The proposed method is novel and addresses research problems in several aspects. First, the method enables researchers to estimate (1) the eyeball size with a spherical model, and (2) the field of view of the retina-RPE in the form of an angle. Thus we can quantitatively investigate if there is a size or shape mismatch between the front and the back of the eye, which provides inferences about eye development, growth, maturation, and emmetropization. To the best of our knowledge, this is the first computational method developed to estimate this field of view angle in the mouse or any other species. This method does not require financial investment or operational skills in professional measurement instruments, including laser micrometer instrumentation. Additionally, this method enables eyeball structural information to be recovered from flatmount images in which no previous eyeball measurements were recorded. For eyeball samples with previous measurements with instruments, this method can serve as a computational tool for a check. As the eyeball measurements with specific instruments must be performed in a timely manner following a regimented protocol, such measuring processes are not forgiving of operator error. Because of the computational nature of this model-based method, it can be invoked repeatedly at any time during or after the course of the experiments.

**Figure 10.**
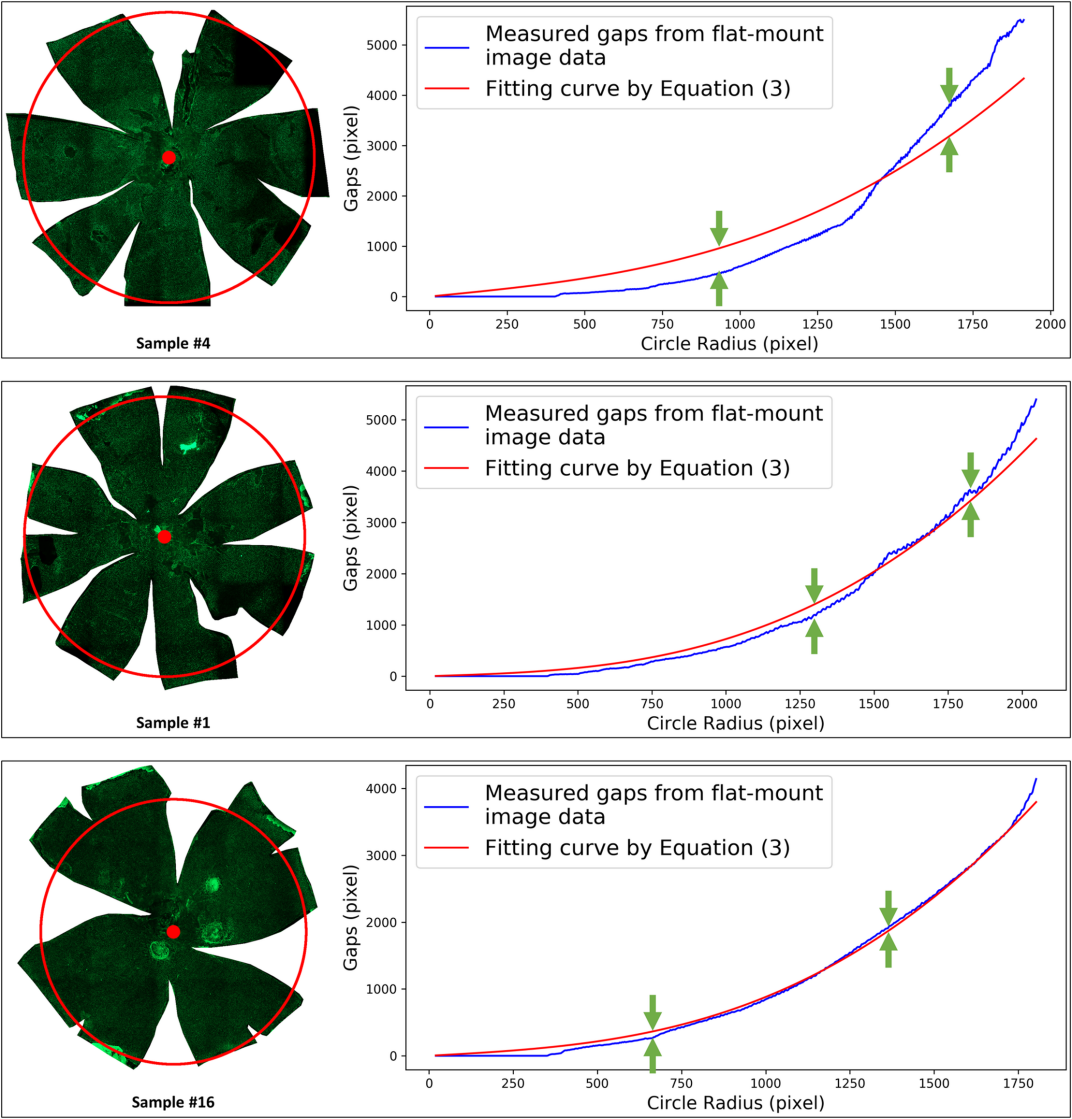
Representative samples with relatively large (top), medium (middle), and small (bottom) differences between measured and fitting interlobe gap curves. Flatmount tissue images of samples 4, 1, and 16 are on the left. Their interlobe gap curve fitting plots are on the right. The blue curve represents the measured gaps derived from flatmount imaging data; the red curve is the fitting curve derived from Equation (3).

The estimation model described in Equation (3) introduced the error-correction term to compensate for the tissue deformation during the eyeball flattening process. With qualitative analysis and careful observations, the distortion error term was assumed to be proportional to sin θ. The quantitative analyses supported the validity of this assumption and indirectly corroborated the tissue distortion analysis. A substantial difference was noted across samples by the variation of the tissue distortion coefficients *k* estimated by distinct data points with some removed from the low-latitude zones, which is shown in Figure 6(c). The difference between the interlobe gaps measured from the flatmount image and the associated fitting curve for this gap indicated the variation of the tissue distortion coefficient *k* estimated with distinct partial data points. Curve fitting plots associated with samples 4, 1, and 16, representing relatively large, medium, and small differences between measured and fitting interlobe gap curves, are shown in Figure 10, respectively. The variations in the tissue distortion coefficient estimated with distinct partial data points were found to be large, medium, and small, respectively, and are shown in Figure 6(a). However, this correlation did not change the underlying trend that exclusion of data points from low-latitude zones led to a decrease in the estimated tissue deformation coefficient. In reference to the ground truth eyeball radius measured with noncontact LED micrometry at submicron accuracy and precision, the proposed method, considering the effects of tissue deformation, achieved 0.03 mm and 5.27% for the average radius difference and the average relative radius difference percentage, respectively.

Note that *k*/2π ranged from 0.05 mm to approximately 0.25 mm, with an average of approximately 0.13 mm. As shown in Table 6, the ground truth value for the eyeball radius ranged from 1.47 to 1.71 mm, with an average of approximately 1.58 mm. By numeric comparisons, the numeric value of *k*/2π was less than *R* by an order of magnitude. As the average *k*/2π was used to correct the estimation error in *R* in Equation (3), the impact of the sample variation in *k* on the radius *R* estimation was limited. In the experiments, the least-squares algorithm was used to estimate *k* and *R* simultaneously. However, this simultaneous approach resulted in inexact estimates of *k* and *R*. Therefore we conclude that it is essential to estimate *k* before *R*.

To assure the effectiveness of the proposed method, we suggest the following sample cutting guidelines that aim to make the resulting flatmount images suitable for the proposed method. (1) The eyeball should be cut radially along the longitudes all the way down to the optic nerve center as much as possible. (2) Each cut should be stopped equally close to the optic nerve center. (3) The resulting lobes should have equal lobe widths. (4) The recommended cutting lobe number is either 4 or 8. In general, a larger lobe number results in better sample flattening, but at the cost of a larger likelihood of tissue loss. Therefore we recommend 4 or 8 lobes as an appropriate tradeoff. For the four-lobe strategy in particular, a small incision cut at the middle of each lobe's distal top is recommended to reduce tension. (5) Poor-quality flatmounts cannot be salvaged with sophisticated analyses. Technicians must practice the flatmounting technique before proceeding. The scissor cuts must be straight and on axis, starting close to the center of the cornea. The RPE surface cannot be scratched. The tissues cannot be overfixed, which results in heavy wrinkling and puckering. Minimized tissue loss or deformation is suggested during the dissection and cutting processes.

The number of lobes is a factor that affects the quantification accuracy. On one hand, a large number of lobes can help reduce the tissue distortion by reducing tissue tension or compression. However, a large lobe number significantly increases the tissue cutting difficulty. As tissues are soft, cutting an eyeball into a large number of lobes presents a challenge to surgical skills. Additionally, a large number of lobes in flatmount practice tend to result in uneven tissue cutting, significant tissue deformation, and even tissue loss problems. These violations of the suggested cutting guidelines, in turn, would introduce greater noise in the image measurements and lead to increased error. On the other hand, the number of cuts cannot be too small. Too few lobes result in wide lobes with severe tissue tension. During the flattening procedure, strong tension within a lobe may lead to tissue tears or severe tissue distortion, both contributing to a larger estimation error. Therefore either 4 or 8 lobes are recommended.

With the estimated eyeball radius, regions of interest from the 2D flatmount image can be mapped to the 3D eyeball surface. Therefore this method can promote development of RPE cell in situ morphology analysis without loss of 3D eyeball structures and facilitate histopathology image analysis for enhanced studies of RPE cell morphometry for eye-related diseases.^6^^,^^21^ Notably, a large number of in situ noninvasive fundus imaging technologies have emerged recently, including adaptive optics optical coherence tomography,^40^^,^^41^ adaptive optics scanning laser ophthalmoscopy,^42^^–^^44^ and fluorescence adaptive optics scanning light ophthalmoscopy,^45^^,^^46^ among others. With advances in these imaging technologies, RPE morphometry analyses become more important in eye disease research, diagnosis, and treatment. The method proposed in this study initiates the effort of constructing a 3D digital eyeball with 2D RPE tissue flatmount microscopy images and makes it possible to leverage image restoration approaches^24^^–^^31^ for such efforts. As the RPE layer is naturally a 3D anatomy structure in a 3D space, it is clinically and biologically meaningful to derive more key information about RPE cell morphometry, orientation, and spatial organization on the 3D eyeball surface. Additionally, the proposed method can be used to facilitate quantitative biometric characterization of mice eyeball size that is related to abnormalities in refractive or ocular development.^32^ Such traits as eyeball volume recovered by the proposed method can also help researchers and clinicians better understand eye growth patterns under certain disease conditions,^47^^–^^50^ the mechanisms vision systems follow to maintain visual clarity,^51^^,^^52^ and key genes affecting visual pathways.^53^^–^^58^

Although the proposed method enables estimation of a 3D eyeball structure from the corresponding 2D flatmount image, it can be further improved in the following ways in the future. (1) The dissection process might be improved by etching or partially eroding the sclera to relax the tissue to reduce wrinkling or bulging. (2) To accommodate the tissue deformation effect during the flatmount procedure, we assume such deformation is related to geographic changes in fiber thickness, orientation, and biomechanical properties of the sclera.^59^ This can be enhanced by development of a better model for characterizing individual tissue deformation specific to a customized tissue flatmount preparation process. (3) The process for identifying the concentric circle center and lobe-marker points can be automated to enable high-throughput image analysis of a large number of flatmount microscopy images. (4) As the anteroposterior axis is slightly different (1%–2% longer) from the sagittal or horizontal axes in some 3D eyeballs,^60^ the 3D geometric sphere for modeling a 3D eyeball may be further improved with a 3D ellipsoid. However, it would be challenging in such methodology development, as we need to simultaneously estimate more parameters in the 3D ellipsoid model, such as the lengths of the three major axes, the polar angle (i.e., the angle respect to the polar axis), and the azimuthal angle (i.e., the angle of rotation from the initial meridian plane). Because of the substantial increase in the number of model parameters for estimation, the resulting method would become much more computationally expensive. Furthermore, given the limited measuring information derived from flatmount images, this change to the ellipsoid model would challenge the estimation robustness. Finally, it is operationally challenging to satisfy the ellipsoid model requirement during the sample preparation procedure. Making the 3D ellipsoid computational method as simple as possible, it is ideal to place the eyeball in a perfectly upright position. However, this is challenging to achieve in practice as eyeballs are made of soft tissues.

## Conclusions

A new method was developed for estimating the 3D eyeball radius and the corneal angle from the 2D flatmount microscopy image, allowing for morphology, orientation, and spatial organization variation analysis of RPE cells in reference to their locations on the original 3D eyeball structure. To compensate for estimation error as a result of tissue deformation during the tissue flatmount production process, we proposed an error-correction term in the model to effectively mitigate the tissue distortion impact. Compared with the ground truth, the proposed method considering the tissue deformation effect achieved 0.03 mm and 5.27% for the average radius difference and the average relative radius difference percentage, respectively. Based on the estimated eyeball radius, it is easy to map tissue regions in a flatmount image to the 3D eyeball surface. This work presents promising potential to promote RPE cell analysis with histopathology tissues for eye disease research, diagnosis, and treatment. To the best of our knowledge, this is the first of such work pioneering the method for reconstructing the 3D eyeball structure from 2D flatmount microscopic images.

## Supplementary Material

Supplement 1

Supplement 2
