# Supplementary material for: Computational Model-Based Estimation of Mouse Eyeball Structure From Two-Dimensional Flatmount Microscopy Images
Source: Transl Vis Sci Technol. 2021 Apr 23;10(4):25. doi: 10.1167/tvst.10.4.25 (PMC8088229; doi:10.1167/tvst.10.4.25)
Supplement: Supplement 1 [file tvst-10-4-25_s001.pdf]

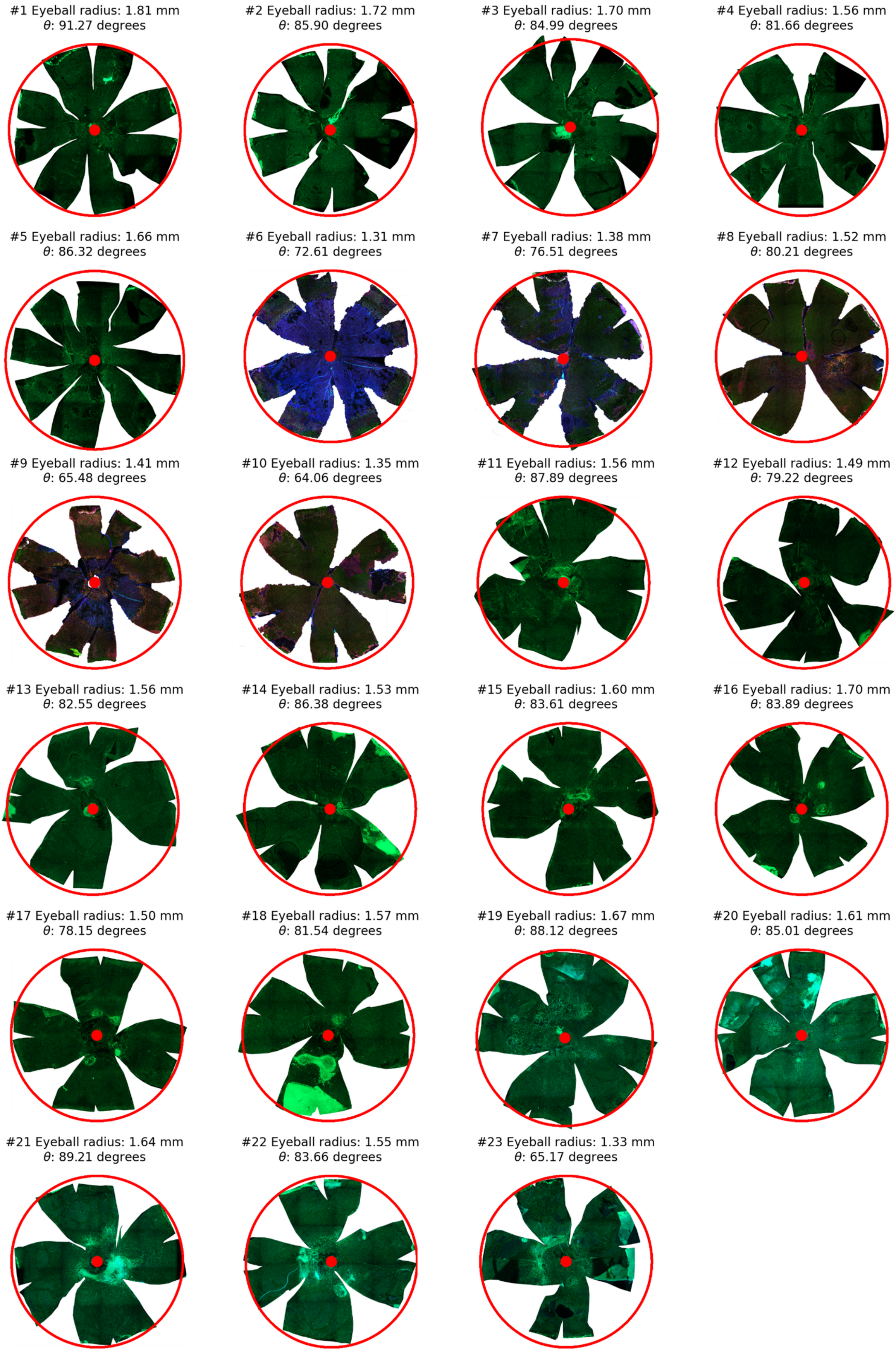

Supplementary Figure 1. Estimates of the eyeball sphere radius and the corneal angle with the model in Equation (3) using the LOO-CV strategy for learning the tissue deformation. The radius and the corneal angle estimated with the model by Equation (3) using the leave-one-out (LOO) cross-validation (CV) are presented. The red spot in the center is the manually annotated origin. The red circle is the concentric circle used to estimate corneal angle θ based on estimated radius R.
