# Supplementary material for: Computational Model-Based Estimation of Mouse Eyeball Structure From Two-Dimensional Flatmount Microscopy Images
Source: Transl Vis Sci Technol. 2021 Apr 23;10(4):25. doi: 10.1167/tvst.10.4.25 (PMC8088229; doi:10.1167/tvst.10.4.25)
Supplement: Supplement 2 [file tvst-10-4-25_s002.pdf]

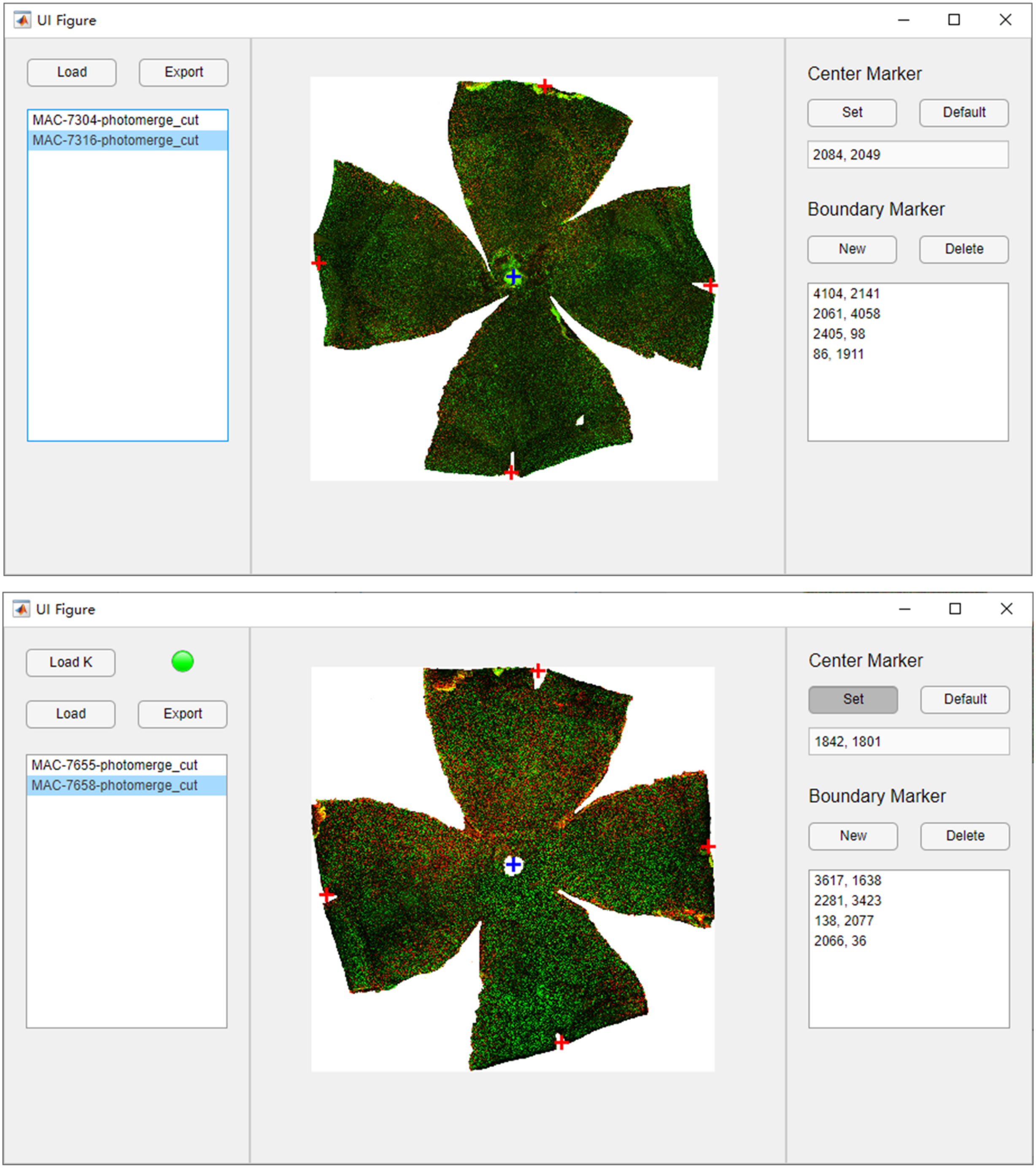

Supplementary Figure 2. Graphical user interface developed to facilitate software use. (Top): The graphical user interface is used to estimate the tissue distortion coefficient. (Bottom): The graphical user interface is used to estimate the eyeball size.
